# *In vitro* fermentation profiles of different soybean oligosaccharides and their effects on skatole production and cecal microbiota of broilers

**DOI:** 10.5713/ab.21.0424

**Published:** 2022-01-05

**Authors:** Xin Zhu, Miao Xu, Haiying Liu, Guiqin Yang

**Affiliations:** 1 College of Animal Science and Veterinary Medicine, Shenyang Agricultural University, Shenyang 110866, China

**Keywords:** Broilers, Gut Microbiota, Odors, Oligosaccharides, Short Chain Fatty Acids

## Abstract

**Objective:**

The objective of this study was to investigate the *in vitro* fermentation profiles of different soybean oligosaccharides (SBOs) and their effects on skatole production and cecal microbiota of broilers.

**Methods:**

Five SBOs with varying main component contents were fermented using an *in vitro* batch incubation inoculated with broiler cecal microbiota. Gas production was recorded automatically, skatole, indole and short-chain fatty acids (SCFAs) were determined using high-performance liquid chromatography, and microbial changes were analyzed using 16S DNA gene sequencing.

**Results:**

The addition of SBOs increased (p<0.05) gas production, suggesting bacterial growth-stimulating activities. In addition, the concentrations of indole were significantly (p<0.05) decreased after SBO supplementation, and SBO III, with higher sucrose and stachyose contents, decreased (p<0.05) the skatole level. Our results also revealed that the fermentation of SBOs by cecal microbiota produced (p<0.05) SCFAs, which were dominated by propionic acid, butyrate acid and lactic acid compared to the control. In addition, SBO III increased (p<0.05) the abundance of Firmicutes and *Subdoligranulum* and decreased that of *Bacteroides*.

**Conclusion:**

These results suggest that SBOs with higher sucrose and stachyose contents are promising prebiotics in modulating gut microbiota and reducing odor emission in broilers.

## INTRODUCTION

Soybean oligosaccharides (SBOs) are a group of soluble oligosaccharides found in soy or other legumes that consist primarily of stachyose, raffinose, and sucrose [1]. SBOs are not usually digested by human or animal digestive enzymes of the upper gastrointestinal tract but are instead selectively fermented by some types of intestinal bacteria in the lower tract [2]. This selective stimulation of a limited number of bacteria affects the host by modulating the microbial community composition and metabolites, such as short-chain fatty acids (SCFAs), in a presumably beneficial way [3]. Dietary supplementation with SBOs has been demonstrated to have prebiotic effects on growth performance, immune modulation and intestinal microbial communities in pigs and broilers [4].

Recently, SBOs were shown to have the ability to lower skatole production [5,6]. Skatole is one of the most noticeable fecal odorants and has a detectable threshold of 0.003 mg/m^3^, thereby having an adverse influence on the productivity and welfare of intensively fed animals [7]. The hindgut is the main site where skatole and indole are mainly produced by the microbial degradation of L-tryptophan (L-Trp) in the diet [8,9]. Previous studies have demonstrated that the addition of SBOs can decrease the rate of L-Trp degradation and the concentration of indole and skatole *in vitro*, in the cecum, and in excreta [5,6,10]. However, the differences in the source and production technology lead to the difference in the spatial structure, thus resulting in the difference in the prebiotic activity of SBOs. Therefore, in this study, an *in vitro* fermentation model was used to investigate the effects of different SBOs with varying main component contents on skatole production and the cecal microbiota of broilers, and the results may contribute to evaluating the prebiotic potential of SBOs to modulate gut health and reduce odor emission in broiler production.

## MATERIALS AND METHODS

### Animal care

All procedures involving animals were reviewed and approved by the Animal Care and Use Committee of Shenyang Agricultural University (201806010).

### Source and composition of soybean oligosaccharides

The SBOs used in the present study were commercially available and supplied by Henan Tiandi Biotech Co Ltd. (SBO I); Henan Fenglu Biotech Co Ltd. (SBO II); Shanxi Jinrun Biotech Co Ltd. (SBO III); Xi’an Baichuan Biotech Co Ltd. (SBO IV); and Guangdong Yibaolai Biotech Co Ltd. (SBO V), China. The functional ingredients in SBOs are sucrose, raffinose and stachyose, and the contents of total sugar (%), sucrose, raffinose and stachyose (mg/g) of SBOs used in this study were determined and shown below: SBO I (66.50%, 66.98, 100.75, 549.79), SBO II (82.75%, 61.82, 114.29, 509.02), SBO III (75.53%, 70.12, 84.70, 559.70), SBO IV (65.54%, 60.36, 109.98, 520.51), and SBO V (70.39%, 77.24, 84.56, 641.44). The moisture content was all less than 5%.

### Animals and cecal digesta collection

Twenty Arbor Acres one-day-old commercial straight run broiler chicks with an average initial body weight were allocated randomly into 2 cages of 10 birds each (male and female in half). Cages equipped with a separate feeder and a nipple drinker from 1 d to 49 d. The basal soybean meal-free diet was formulated to exclude the interference factor of oligosaccharides from soybeans and to meet the nutrient requirements recommended by the Arbor Acres management guide (Table 1). On day 49, broiler chicks were sacrificed, abdominal cavities were opened immediately, and both ceca of each chick were collected aseptically, tied from open sides, placed into empty sterile plastic bags individually and stored at −80°C.

### Experimental design

A single factor complete random design was adopt in this study. A total of six treatments were included: one control and five SBO treatments (SBO I to SBO V). The total volume of the fermentation broth was 200 mL each, and 1.5% SBO was contained in a treatment on a total sugar basis and L-Trp was added to each treatment at a final concentration of 250 μmol/L. During each incubation, no L-Trp addition was used for adjustment for gas and fermentation metabolite production. The incubation temperature and time were 41°C and 24 h, respectively. Each treatment was carried out in 3 technical repetitions.

### *In vitro* fermentation

The basal salt medium was formulated according to Wang et al [11] and then autoclaved (121°C, 15 min) for sterilization [11]. The mixed cecal digesta (male:female = 1:1) were suspended under a constant flow of CO_2_ gas in sterile basal salt medium to give a final concentration of 100 g of digesta/L and homogenized in a blender. A volume of 198 mL of the suspension was added to a fermenter autoclaved before use, and 2 mL of L-Trp solution was added to give a final concentration of 250 μmol/L. As a control, a fermenter with 198 mL of the suspension and 2 mL of sterilized distilled water was used to measure the cecal contribution to the production of gas, skatole, indole, and SCFA. The incubation was kept at 41°C for 24 h. Samples (10 mL) were taken after incubation for analysis.

### Gas production

The ANKOM-RFS gas production automated system (GPM 9.8.1) (ANKOM Technology, Macedon, NY, USA) was used to measure gas production following the equation:


Vxt=Vj×(Ppsit-Ppsi0)×0.068

where Vxt (mL) is the cumulative gas production at time t (h) during fermentation, Vj (mL) is the headspace of the glass bottles, Ppsit is the cumulative pressure (psi) of the sample modules and Ppsi0 is the psi of the blank module at time t as recorded by the system.

### Skatole and indole analysis

The skatole and indole concentrations were analyzed by the high-performance liquid chromatography (HPLC) method as described previously [10]. The HPLC conditions were as follows: column, 250 mm×4.6 mm×5 μm (Dikma, Beijing, China); eluent, acetonitrile/water (40:60, v/v); flow, 1.0 mL/min; excitation wavelength, 263 nm; emission wavelength, 358 nm; injection volume, 20 μL.

### Volatile fatty acid and lactic acid analysis

The volatile fatty acid and lactic acid concentrations were analyzed by HPLC method as described previously [10]. The HPLC conditions were as follows: column, 250 mm×4.6 mm× 5 μm (Dikma, China); eluent, phosphate buffer (pH 2.5)/methyl alcohol (95:5, v/v); flow, 1.0 mL/min; injection volume, 10 μL.

### DNA extraction and polymerase chain reaction amplification

Total genomic DNA from the fermentation broth samples was extracted using a Qubit 2.0 DNA kit according to the manufacturer’s instructions. 16S rDNA genes of V3–V4 were amplified using 341F-805R (341F: 5′-CCCTACACGACGC TCTTCCGATCTG-3′; 805R: 5′-GACTGGAGTTCCTTG GCACCCGAGAATTCCA-3′) with the barcode as described by Liu et al [6]. The polymerase chain reaction (PCR)-amplification products were verified by agarose gel electrophoresis (1.5% agarose).

### Bioinformatics analysis

A total of 18 cecal fermentation samples were collected and sent for sequencing. Raw reads were quality filtered to remove primer connector sequences, nonamplification sequences, or chimeric sequences [12]. The remaining high-quality reads with sequence lengths of 400 to 440 bp across all the samples were processed, merged and clustered into operational taxonomic units based on a 97% sequence similarity threshold. The species annotation and statistical analysis of the species composition for each sample at the phylum and genus levels were performed by the Ribosomal Database Project classifier software and the Greengenes database [13]. Microbial community structure analysis was included for the observed species and the alpha diversity indices Chao1 index and ACE index, which were used to estimate species richness, Shannon index and Simpson index, were used to estimate species diversity [14]. Principal coordinates analysis and cecal microbiota cluster trees, which revealed the similarity measure of bacterial community based on phylogenetic distance, were performed based on unweighted UniFrac distance matrices.

### Statistical analysis

Statistical analysis was performed by one-way analysis of variance using SPSS 17.0 software (SPSS Inc., Chicago, IL, USA), and significant differences among treatments were compared using Duncan’s multiple comparison tests [10]. Differences with p-values less than 0.05 were considered statistically significant.

## RESULTS

### Gas production

**Figure 1:**
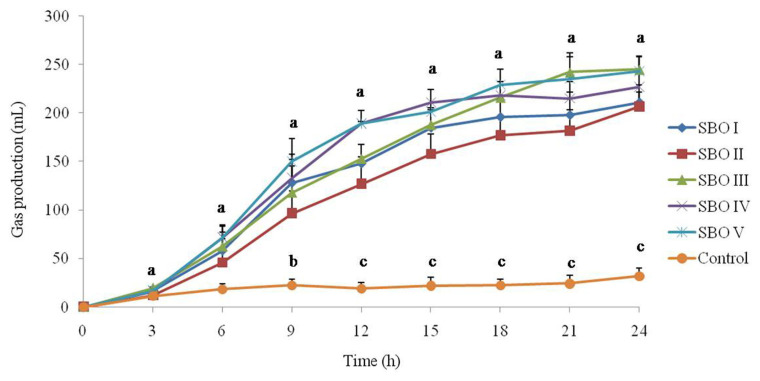
Effect of different soybean oligosaccharides (SBOs) on gas production in cecal fermentation broth from broilers. Different letters indicate significant differences among treatments (p<0.05).

The cumulative gas production is shown in Figure 1. All tested SBOs produced gas when they were utilized by cecal microbial communities. Gas production in the control was low at all sampling times, while the addition of SBOs increased (p<0.05) gas production after 9 h of incubation and even more significant (p<0.01) after 12 h of incubation. Among SBOs, SBO II had a lower (p<0.05) cumulative gas production than SBOs IV and V after 12 h of incubation.

### Skatole and fermentation parameters

**Figure 2:**
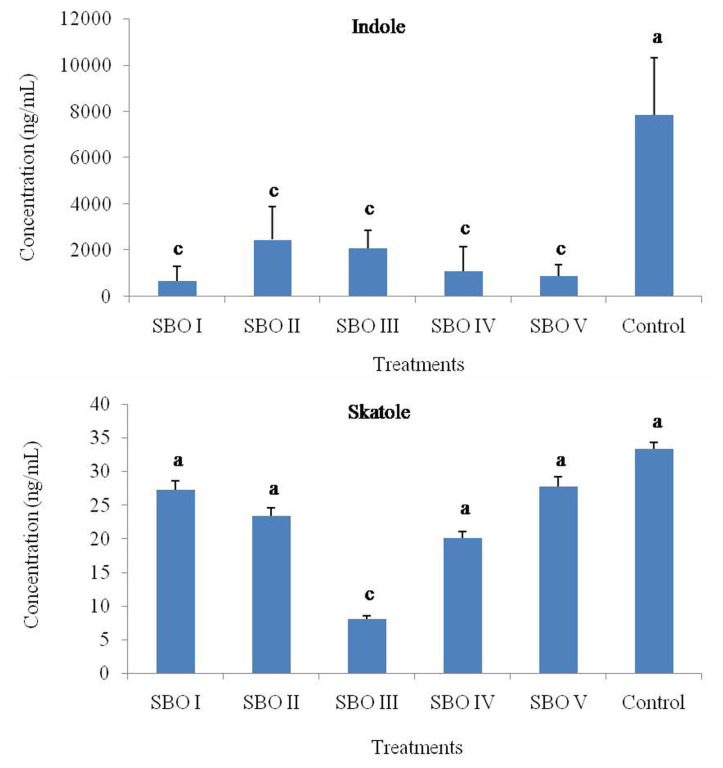
Effects of different soybean oligosaccharides on indole and skatole concentrations in cecal fermentation broth from broilers. Different letters indicate significant differences among treatments (p<0.05).

Skatole concentrations showed no significant difference (p> 0.05) among treatments except SBO III, which had a lower (p<0.01) skatole level. Compared to the control, indole concentrations of SBOs were lower (p<0.01) (shown in Figure 2).

**Figure 3:**
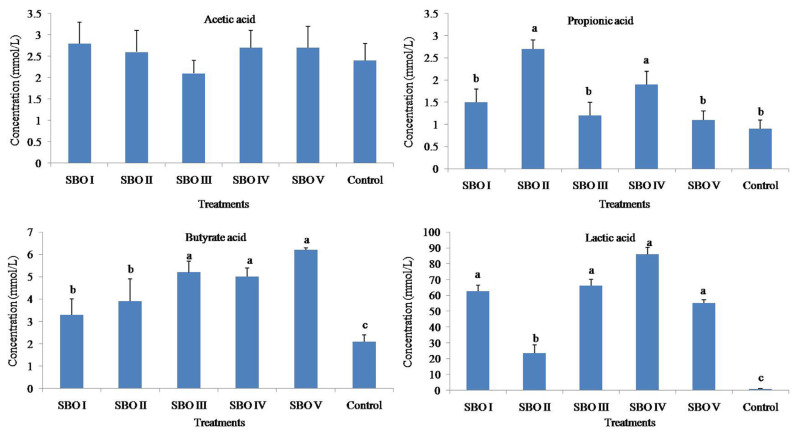
Effects of different soybean oligosaccharides (SBOs) on SCFA concentrations in cecal fermentation broth from broilers. Different letters indicate significant differences among treatments (p<0.05).

The addition of SBOs increased (p<0.01) the concentrations of propionic acid, butyrate acid and lactic acid but did not (p>0.05) affect that of acetic acid. The butyrate acid concentrations in SBOs III, IV, and V were higher than those in SBOs I and II (shown in Figure 3).

### Microbial community structure

**Figure 4:**
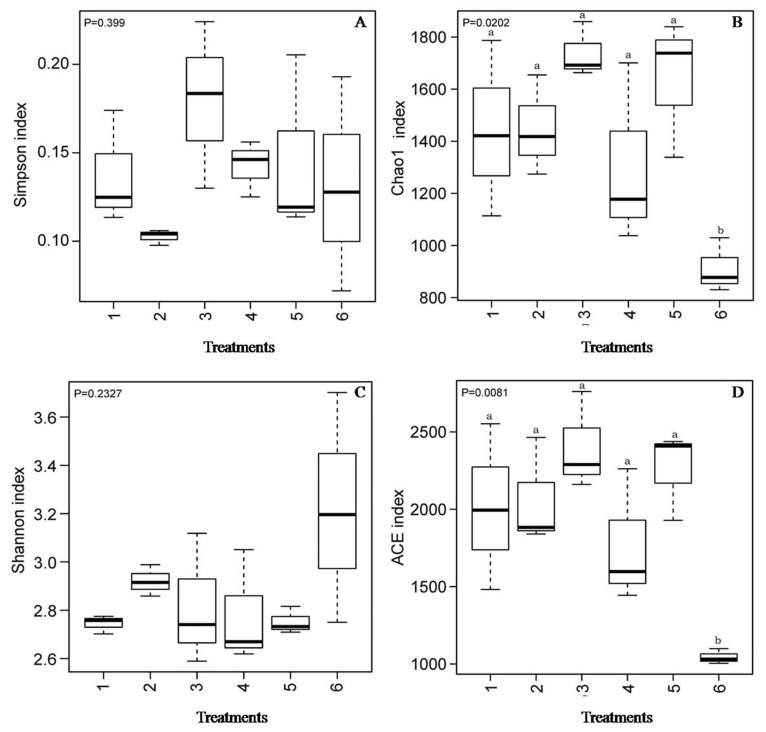
Alpha diversity index of cecal microbiota in fermentation broth from broilers. A, Simpson index; B, Chao1 index; C, Shannon index; D, ACE index. Treatment 1 to 5, SBO 1 to 5; Treatment 6, control.

**Figure 5:**
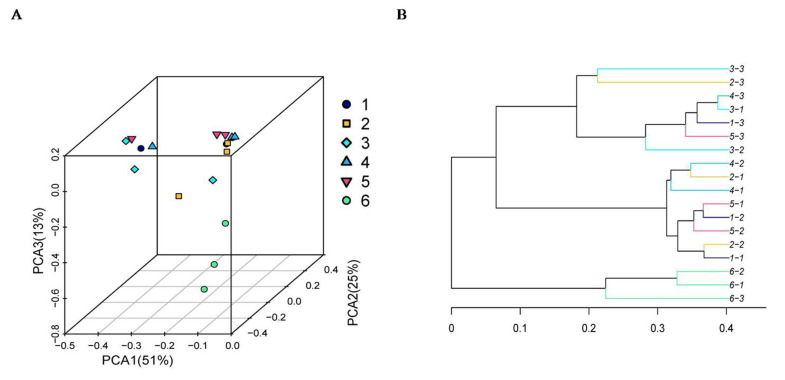
Principal coordinate analysis (A) and cluster tree diagram (B) of cecal microbiota in fermentation broth from broilers. Treatment 1 to 5, SBO 1 to 5; Treatment 6, control.

As shown in Figure 4, the Chao 1 index and ACE index of the SBO treatments were higher (p<0.01) than those of the control. However, there was no statistical difference (p> 0.05) in the Shannon index and the Simpson index among treatments, although the Shannon index of the control was numerically higher than that of the SBO groups. The results of the cluster trees showed a degree of diversity discrepancy between the SBO III group and the control, while the samples from other SBO treatments clustered together (Figure 5).

**Figure 6:**
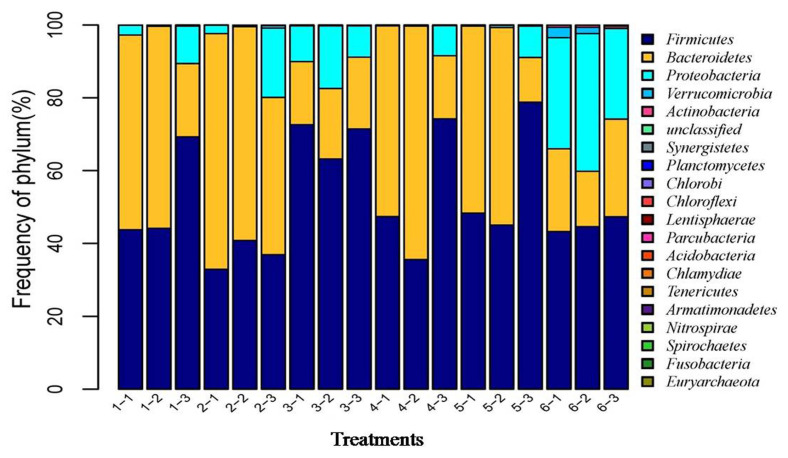
Effects of different soybean oligosaccharides (SBOs) on cecal microbiota in fermentation broth at the phylum level. Treatment 1 to 5, SBO 1 to 5; Treatment 6, control.

**Figure 7:**
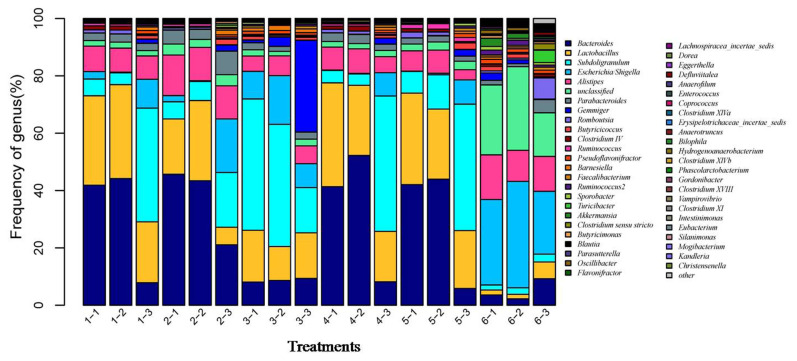
Effects of different soybean oligosaccharides (SBOs) on cecal microbiota in fermentation broth at the genus level. Treatment 1 to 5, SBO 1 to 5; Treatment 6, control.

After the microbial diversity analysis, the taxonomic profiles of the fermentation samples were explored. As shown in Figure 6 and 7, seven phyla and twenty-three genera with relative abundances greater than 0.1% were present. At the phylum level, Firmicutes and Bacteroidetes were prevalent among all treatments but were different in the control and SBO treatments. It is worth noting that Bacteroidetes were decreased in SBO III compared with the other SBO treatments, whereas Proteobacteria increased (p<0.01). At the genus level, *Bacteroides* and *Lactobacillus* were present in all treatments. Compared with the control, the addition of SBOs decreased the abundance of *Escherichia*/*Shigella*, *Alistipes*, *Sporabacter*, *Ruminococcus2* and *Butyricumonas* and increased that of *Ruminococcus*. The results also showed that *Bacteroides* and *Ruminococcus* were less abundant and *Subdoligranulum* was more abundant in SBO III than in the other SBO treatments.

## DISCUSSION

Odorous compounds from livestock and poultry systems are mainly produced from the microbial fermentation of undigested nutrients of the diet in the gut [15]. In poultry, skatole and indole are produced from a multistep degradation of L-Trp by microbial activity and are considered to be contributors to excreta malodor [16]. In addition, the concentrations of skatole and indole in the cecum are significantly higher than those in the other gut segments [9]. Therefore, the cecal digesta of broilers were collected and used as the fermentation medium to determine skatole and indole production in this study.

Oligosaccharides have been reported to possess prebiotic activities and can be utilized by the cecal microbial communities of broiler chickens [17]. During the fermentation process, oligosaccharides can be fermented to produce gases and SCFAs, which are ensemble products of the activities of the total microbiota present in fermentation [2,18]. Thus, gas and SCFA production are one of the most visual indices to evaluate the fermentation state and microbial activity. Along with gas production, SCFAs, including acetic acid, propionic acid, butyric acid and lactic acid, are also produced, which provide important carbon sources for microbial protein synthesis and energy for intestinal cells [3,19,20]. The cumulative gas and SCFA production results in the present study showed that the addition of SBOs had a strong ability to increase gas and SCFA production, implying that SBOs were much easier to utilize by cecal microbes of broiler chickens. In addition, we also observed that fermentation of different SBOs gave rise to varied gas and SCFA production. Presumably, the reason for this may be the varied content and composition of components in SBOs. As the main components of SBOs, raffinose contains fructose, glucose, and galactose, while stachyose contains one more galactose [1]. Different SBOs with varied degrees of polymerization can cause varied production of gases and SCFAs and fermentation profiles. Lan et al [2] compared *in vitro* gas production for SBO, stachyose and raffinose and found that stachyose had higher gas production and maximum rate of gas production than SBO and raffinose [2]. Similarly, in this study, SBOs with a lower content of stachyose (such as SBO II) had a lower gas production, implying that stachyose was a key component of SBOs to be correlated with gas production. In addition, Lan et al [2] also found that stachyose had a stronger ability to induce propionic acid production, but not acetic acid or butytic acid, than raffinose *in vitro* fermentation. Zhu et al [10] also found that dietary supplementation with 0.6% stachyose significantly increased the propionic acid concentration in the cecum of broilers, and 0.6% raffinose seemed to contribute to butyric acid and lactic acid production. Thus, the addition of SBOs can increase the amount of fermenable carbohydrates, stimulate the growth of specific bacteria, and lead to gas and SCFA production.

Skatole is a well-known, foul-smelling fecal odorant affecting the production and welfare of farm animals [7]. Dietary supplementation with some oligosaccharides, such as SBOs, contributes to the modulation of intestinal microbiota by simulating the proliferation of beneficial strains and inhibiting the growth of pathogenic bacteria and then reduces the production of odor compounds [5,10,21]. Liu et al [6] found that the *in vitro* addition of SBO to fermentation broth at a content of 10 g/L total sugar decreased the rate of L-Trp degradation and the concentration of skatole and indole. The components of SBOs, such as stachyose and raffinose, have been demonstrated to affect the production of skatole and indole. Zhu et al [10] also demonstrated that dietary supplementation with 0.6% stachyose significantly decreased the cecal concentration of skatole compared with raffinose in broilers. The results from Liu et al [22] further demonstrated that broiler cecal skatole levels can be influenced by dietary stachyose levels, and 5 g/kg stachyose in the diet was suggested. Similarly, in this study, SBOs with higher sucrose and stachyose contents had a stronger ability to lower skatole and indole production, suggesting that the addition of SBOs may stimulate the growth of carbohydrate-degrading bacteria, not protein-degrading bacteria, and inhibit protein decomposition, thus decreasing the L-Trp flow supply and resulting in reduced skatole and indole production.

In general, the intestinal microbiota of the host contains trillions of symbiotic microorganisms and remains quite stable once established [23,24]. There are many factors, including diet, breed type, and housing environment, which can affect the gut microbiota [25,26]. Of these, diet is the principal environmental factor that can directly influence the nature of the microbiota in the host. Previous studies have demonstrated that dietary supplementation with prebiotics, such as SBOs, which have received much attention after phasing out antibiotic growth promoters in poultry feed, can be selectively fermented and has been linked to the selective growth promotion of probiotics, chiefly *Bifidobacterium* and *Lactobacillus*, thus offering competitive colonization resistance against pathogenic bacteria [5,10,27]. Previous studies have demonstrated that *in vitro* or dietary addition of oligosaccharides resulted in an increase in microbial diversity and species richness [4,21,22]. Similarly, our data also showed that the addition of SBOs increased the microbial diversity and species richness in fermentation broth, indicative of stimulating the growth of microorganisms by SBOs. Significant differences in the populations of bacteria at the phylum level were observed. As a dominant bacterial phylum in the gut microbiota, Firmicutes are always abundant in the lower gut and are capable of degrading oligosaccharides into SCFAs [28,29]. For Bacteroidetes, which are generally considered primary saccharolytic bacteria capable of degrading an extensive array of complex carbohydrates [30], the relative abundance in SBOs was higher than that in the control. At the genus level, *Lactobacillus* were strongly stimulated to proliferate by the addition of SBOs, in agreement with previous results showing an increase in *Lactobacillus* in broilers fed SBOs [5]. Although all SBOs can be degraded by complex gut microbiota, the emergence of high relative abundance bacteria in different SBOs varied. *Bacteroides* was less abundant in SBO III than in other SBO treatments and correlated with reduced skatole production, which is in agreement with previous results showing that *Bacteroides* can metabolize tryptophan to indole-3-lactate and then to indole and skatole [10].

In conclusion, the present work provides evidence on the *in vitro* prebiotic potential of the different SBOs and can be beneficial to gut health. The results indicated that the addition of SBOs can selectively stimulate the growth and activity of beneficial bacteria, increase propionic acid, butyrate acid and lactic acid levels and reduce skatole and indole production in broilers. Moreover, the microbial community structure was substrate-dependent with SBOs, and SBO III, with higher sucrose and stachyose contents, markedly impacted the relative abundance of *Bacteroides* and *Subdoligranulum*. This finding might point out a potential relationship between the main components of SBOs and their impact on the gut microbiota composition and odor compound production.

## Figures and Tables

**Table 1:** Ingredient and chemical composition of the basal diet (air dry basis, %)

Items	Content	

	0 to 3 wk	4 to 7 wk
Ingredients		
Corn	41.40	47.32
Corn umbilicus pulp	23.36	16.00
Corn protein	18.70	15.16
Soybean oil	3.54	4.34
Corn DDGS	3.00	5.00
Rice bran	5.00	8.00
Limestone	1.20	1.14
CaHPO_4_	1.42	1.05
NaCl	0.26	0.27
Lys	1.00	0.84
Met	0.24	0.20
Thr	0.22	0.17
Arg	0.45	0.30
Phytase	0.01	0.01
Choline chloride	0.05	0.05
Vitamin mix^1)^	0.05	0.05
Mineral mix^2)^	0.10	0.10
Total	100.00	100.00
Calculated values		
ME (MJ/kg)	12.77	13.39
CP	22.00	19.50
EE	6.89	8.31
Ca	0.92	0.79
Total P	0.67	0.62
Available P	0.36	0.30
Available Lys	1.30	1.12
Available Met	0.65	0.57
Available Met+Cys	0.90	0.80
Available Thr	0.93	0.80

DDGS, dry distillers grains with solubles; ME, metabolizable energy; CP, crude protein; EE, ether extract; Ca, calcium; P, phosphorus.

1) Contained 22,500 IU/kg vitamin A; 5,500 IU/kg vitamin D_3_; 35 IU/kg vitamin E; 5 mg/kg vitamin K_3_; 2.5 mg/kg vitamin B_1_; 1.5 mg/kg vitamin B_2_; 20 mg/kg vitamin B_6_; 5 mg/kg pantothenic acid; 2 mg/kg folic acid; 75 mg/kg niacin; 0.12 mg/kg biotin; and 0.25 mg/kg antioxidant.

2) Contained 60 mg/kg Mn; 44 mg/kg Fe; 76.5 mg/kg Zn; 6.8 mg/kg of Cu; 10 mg/kg K; and 14 mg/kg Na.
